# Factors contributing to the lack of interest in research activities among postgraduate medical students

**DOI:** 10.12669/pjms.344.15411

**Published:** 2018

**Authors:** Imran Saeed, Nabiha Farasat Khan, Attia Bari, Rehan Ahmed Khan

**Affiliations:** 1 Dr. Imran Saeed, FCPS, MHPE. Associate Professor of Pediatric ENT, Department of ENT &Paediatric Medicine, The Children’s Hospital and The Institute of Child Health, Lahore, Pakistan; 2 Dr. Nabiha Farasat Khan, BDS, M. Phil, MHPE. Associate Professor and Head of Department Oral Pathology, Dental Section, Dental Section Bolan Medical College, Quetta, Pakistan; 3 Dr. Attia Bari, D.C.H, M.C.P.S, F.C.P.S. MHPE. Associate Professor of Pediatric Medicine, Department of ENT &Paediatric Medicine, The Children’s Hospital and The Institute of Child Health, Lahore, Pakistan; 4 Prof. Rehan Ahmed Khan, MBBS, FCPS, FRCS, JM-HPE, M.Sc. HPE. Professor of Surgery, Islamic International Medical College, Riphah International University, Islamabad, Pakistan

**Keywords:** Factors, Knowledge, Perceived, Research, Resident

## Abstract

**Objective:**

To determine the factors contributing to lack of interest in research activities among postgraduate residents in pediatric medicine.

**Methods:**

This cross-sectional survey was conducted at The Children’s Hospital Lahore, Pakistan in August 2017. The questionnaire was distributed to 105 postgraduate residents working in pediatric medicine department and 90 of them returned the completed proforma (response rate; 86%). Data was analyzed by SPSS version 22.

**Results:**

Out of 90 residents’ females were predominant (n=58/90; 64.4%). Mean age of the participants was 28.22 ± 2.092 years. Majority were unmarried (n=57; 63.3%). The highest mean score (2.31±0.697) was regarding “*Lack of proper training for research”*, followed by “lack of previous exposure” (2.26±0.728) and “over loaded curriculum” (2.13±0.753). “Uncooperative faculty” and “funding issues” as a barrier towards research (p=0.016 and 0.014 respectively) was mentioned by males more than females. “Social and family commitment” was a significant perceived barrier in married residents as compared to unmarried residents (p=0.001). The residents in the younger age group were more reluctant to do research due to “over loaded curriculum” (p=0.038).

**Conclusion:**

Lack of proper training of research, lack of previous exposure and time management are the major factors affecting resident’s research work, whereas married residents face more social/family constraints as compare to unmarried residents.

## INTRODUCTION

Research assists in constructing students to be a critical thinker, enhances problem solving and decision making abilities; in addition it furnishes high quality health care services.^1^,^2^ Researchers are able to update their knowledge and skill according to the latest approaches in medicine and have the opportunities to explain things concerned with cognition acquisition and performance, and provides new interpretations of existing facts.^3^ Research is an essential part for any educational system; it makes medical doctors richer in thoughts and updates medical students on the latest advances in medicine.^4^

Extensive literature search pointed out some barriers which play major role in reducing students involvement in research.^2^ One major factor noticed was lack of trained physicians/ scientists.^5^ However, results of other studies discovered that majority of residents did not find any interest in research due to their family commitments and lack of awareness of research methodology.^6^-^8^ Students are not trained to review the literature and they face the problem of unavailability of mentors and non-cooperative supervisors also.^9^ Campbell found imperfect clinical research activities and his results demonstrate major threats present in health centers.^10^ Many diverse factors have been attributed in this regard and knowing these factors may help to find solution for these problems.^3^

Although Pakistani students are keen to study and have positive knowledge and attitude towards health research,^4^ but they are unable to progress in this field due to multiple factors.^11^ Under and post graduate students show lack of interest in research activities^3^, as they face many problems to conduct research, including lack of research knowledge, unavailability of mentors and funds and time management.^3^,^11^-^14^ So there is a need to investigate the leading components that are affecting the student’s research activities. This study was therefore carried out with the aim to find main factors contributing to the lack of interest in research activities among postgraduate medical students at The Children’s Hospital, Lahore.

## METHODS

A cross-sectional survey was conducted in August 2017 through self-administer questionnaire at The Children’s Hospital and The Institute of Child Health (ICH) Lahore, Pakistan which is a tertiary care hospital with 11,00 beds. Ethical approval was taken from the Institutional Review Board of ICH. The themes of all questions used in the current study were taken from Sheikh and Amin.^3^,^15^ After taking informed consent from all PG trainees of pediatric medicine department, questionnaire was distributed to the residents by colleagues of chief researcher.

Before starting the study, questionnaire was pilot tested on 20 PG trainees working at the ICH. The reliability of the questionnaire was 0.725 Cronbach’s alpha. Questionnaire was distributed to 105 PG trainees of both genders working at ICH in addition to the 20 PG training who contributed to the pilot study. Ninety postgraduate residents returned the completely filled questionnaire with the response rate of 86%.Data from the questionnaire were entered in the SPSS software (version 22) and the results were generated. Respondent’s perceptions about barriers to medical students’ research were measured through questions or statement based on Likert scale responses. Likert scale description was: 1=Neutral, 2=Agree and 3=Strongly agree.

The frequencies and percentages were calculated for age, gender, year of training and marital status. The mean scores and standard deviations were calculated for all items separately. The scores were then compared among the participants from different age groups, genders and marital status to see the difference. Mann Whitney U test was applied to find the significant differences of each of the demographic variables under study, i.e. gender, marital status and age. Significance of difference was determined using p-value, which was considered statistically significant at ≤0.05.

## RESULTS

There were 90/105 residents who returned completed questionnaire with an overall response rate of 86%. Majority 64.4% of the participants (n=58/90) were females. Age of the participants ranged from 25 to 33 years (Mean age; 28.22 years ± 2.092). Only 26 (29%) were > 30 years of age. Majority 57 (63.3%) were unmarried.

The question with highest mean score was question number 4 that is *“Lack of proper training for research”* (2.31 ± 0.697), which indicates the majority of the participants were of the view that there is gap of proper training and knowledge of research. Whereas second highest average score was gained for the statement “*Lack of pervious exposure”* was 2.26 ± 0.728 (CI 2.10-2.41) which shows that most of them perceived that as they never had an experience of research so previous experience matters a lot and this was a major barrier in their future research interest. Similarly, mean score of question 6 “*Inaccessibility to Electronic data”* and question 7 “*Inaccessibility to hospital register”* was 1.63±0.644 (CI 1.50-1.77) and 1.67±0.670 (CI 1.53-1.81) respectively skewed towards neutral indicating that inaccessibility to electronic data and hospital registers was not a barrier in their research interest. The mean person score regarding different factors is shown in (Table-I).

**Table-I:** Distribution of residents’ perceptions of barriers towards research.

Q. No	Perception of residents about barriers towards research	Mean Score & SD (95%CI)	All PGs Agreement Score		

			Neutral	Agree	Strongly Agree
1	Lack of Time	2.07±0.650 (1.93-2.20)	17.8%	57.8%	24.4%
2	Lack of Motivation	2.08±0.738 (1.92-2.23)	23.3%	45.6%	31.1%
3	Social/Family commitments	1.81±0,763 (1.65-1.95)	40%	38.9%	21.1%
4	Lack of proper training of research	2.31±0.697 (2.17-2.46)	13.3%	42.2%	44.4%
5	Lack of interest	1.66±0.721 (1.50-1.81)	48.9%	36.7%	14.4%
6	Inaccessibility to Electronic data	1.63±0.644 (1.50-1.77)	45.6%	45.6%	8.8%
7	Inaccessibility to Hospital Register	1.67±0.670 (1.53-1.81)	44.4%	44.4%	11.2%
8	Lack of research Knowledge by Faculty	1.96±0.686 (1.80-2.30)	25.6%	53.3%	21.1%
9	Lack of research Knowledge by PGs	1.94±0.675 (1.80-2.09)	25.6%	54.4%	20.0%
10	Lack of proper internet facilities/opportunities	1.69±0.774 (1.53-1.85)	50.0%	31.1%	18.9%
11	Lack of proper Laboratory facilities	1.77±0.750 (1.61-1.92)	42.2%	38.9%	18.9%
12	Uncooperative Faculty	1.78±0.804 (1.61-1.95)	45.6%	31.1%	23.3%
13	Lack of Funding	2.00±0.807 (1.83-2.17)	32.2%	35.6%	32.2%
14	Finding Supervisor	1.84±0.763 (1.68-2.00)	37.8%	40.0%	22.2%
15	Searching Topic	1.99±0.711 (1.84-2.14)	25.6%	50.0%	24.4%
16	Over Loaded curriculum	2.13±0.753 (1.98-2.29)	22.2%	42.2%	35.6%
17	Lack of pervious exposure	2.26±0.728 (2.10-2.41)	16.7%	41.1%	42.2%
18	Uncooperative community/samples	1.91±0.816 (1.74-2.08)	37.8%	33.3%	28.9%

The top three factor hindering PG trainees research activities based on agree and strongly agree response were “*lack of proper training of research”* (86.6%),”*Lack of pervious exposure”*(83.3%) and “*Lack of Time”*(82.2%).Other significant factors to be counted as hurdle in research work of PGs include “*overloaded curriculum”* (77.8%) and “*lack of motivation”* (76.7%).The frequency and percentages who agreed or strongly agreed is shown in (Table-I).

The study result indicates that PG trainees who were married had more social/ family commitments as 84.8% married doctors agreed with this question as compared to unmarried colleagues 45.6% (p=0.001) The significant differences based on age groups and gender are shown in (Table-II).

**Table-II:** Difference in the perception of residents according to age and gender.

Q. No	Perception Questions	Neutral	Agree	Strongly Agree	Neutral	Agree	Strongly Agree	p-value

*Age groups*		25-30 Years			>30 Years			
3	Social/Family commitment	48.4%	35.9%	15.6%	19.2%	46.2%	34.4%	0.022
16	Over load curriculum	18.8%	37.5%	43.8%	22.2%	42.2%	35.6%	0.038
17	Lack of previous exposure	10.9%	40.6%	48.4%	30.8%	42.3%	26.9%	0.041

**Gender**		**Male**			**Female**			

12	Uncooperative Faculty	34.4%	25.0%	40.6%	34.5%	34.5%	13.8%	0.016
13	Lack of Funding	15.6%	53.1%	31.2%	41.4%	25.9%	32.8%	0.014

## DISCUSSION

Research is crucial in health profession as it furnishes proficient guidance and acquisition of latest updates.^16^ In our study, a total of 105 residents included in the study working at the ICH Lahore, 90 residents returned completed questionnaire. Overall response rate was 86%. Same response rate was observed in Abushouk analysis where the questionnaire was filled up by 86% medical students.^17^ However the results of Amin^15^ demonstrated high response rate (91%), whereas lowest response rate 63.1%was reported in Karraz study done in Kuwait University where 64.8% students participated in the study. 2Age of the participants ranged from 25 years to 33 years (mean age = 28.22 ± 2.092years). The mean age of Alghamadi research was 23±1.17years.^18^Undergraduate students were selected for enquiring the factors for lack of interest in research in these studies as compared to postgraduate trainees in our study which resulted in increased in mean age of participants in our study.

The mean score for statement *“Lack of time”* was 2.07±0.650 (CI; 1.93-2.20), indicating that most of them show agreement just due to their educational commitments. Results of two studies conducted by Alghamadi and other by Karraz revealed lack of time as the top most barriers in research activities as 72.3% and 77.4% participants face difficulty in managing time for research procedures and majority of them were females in the study by Karraz.^2^,^18^ Same factor of shortage of time was reported by Bilquees.^19^

If residents are married they require fostered social and emotional support from their spouse.^20^ Although marital status affects educational life of persons but this demographic view was not considered by other researchers. In our study, marital status of the postgraduate trainee resulted in their increased family commitments hindering their research activities. However, if the spouse is cooperative, educated and supports emotionally and socially it reduces the burden and enhances academic performance of female medical students.^20^

“*Lack of proper training/knowledge of research*” achieved highest mean score 2.31 ± 0.697. High score gaining question indicates that majority of the participants have the view that there is gap of proper training and knowledge of research.^21^ A large percentage of 88.8% participants of Alghamadi agree that they are not trained properly for research activities,^18^ likewise 71.6% medical students of Mostafa study agreed about deficient research knowledge.^14^ Various studies from all over the world showed similar results showing lack of training as hindering factor towards research.^3^,^15^,^16^

*“Overloaded curriculum”* accounted for mean score 2.13±0.753 (CI; 1.98-2.29). Sheikh in his research observed 47.1% cases and 52.9% controls feel overloaded curriculum to be a hurdle in research activities.^3^ In other countries including Pakistan research is not a part of curriculum and the course is overloaded due to traditional teaching and learning strategies, thus, students found it difficult to overcome this problem and are unable to start their research activities during their professional educational life.^3^,^14^,^19^ In our study postgraduate trainees in early years were more over burdened by heavy curriculum as compared to senior trainees (p=0.038).^3^,^14^,^19^

The average score for “*lack of pervious exposure”* was 2.26 ± 0.728 (CI 2.10- 2.41) which shows that most of them perceived that they never had an experience of research. So, previous experience matters a lot and this was a major barrier in their future research interest. A study by Sheikh et al showed similar findings in which residents agreed with facing the problem of lack of previous exposure of research activities.^3^

Teacher or faculty mentoring is crucial for the youngsters during the journey of their educational life. Mentoring during research is effective in the selection of mentees’ carrier in their choice of specialty.^22^ Kharraz research revealed that 70% students find it difficult to approach a mentor.^2^ Whereas 78.3% females and 67.7% males of Abushouk study participants complained of uncooperative mentor.^17^ In our study 62.2% residents assume lack of mentoring. As mentor reinforces and assists in facilitating a trainee’s education; he/she also accelerates trainee’s acquisition of research skills and career development so participants perceive its adverse effect on their research work.

Limitations

Self-reported questionnaire was utilized which results in respondent bias and the response rate may result in possible selection bias. It has been undertaken only in one medical institute and in one department so limiting the generalization of our results. There is need for multi-centric studies.

## CONCLUSION

The issues that hinder research activities of medical students are lack of proper training of research, lack of previous exposure, lack of time and overloaded curriculum. Barriers including difficulty in finding a mentor can be removed by assigning faculty for students working as mentors and supervisor. Curricular inadequacy can be resolved by its revision and including research methodology in it.

### Recommendations

Research activities should be mandatory for graduation which helps students understanding skills and process of research.

### Author’s contribution

**IS:** Conceived idea.

**NFK:** Manuscript writing.

**AB:** Data collection, data analysis, contributed in manuscript writing, final review and editing.

**RAK:** Critical review, suggestions and final approval.
